# Detecting treatment response in a model of human breast adenocarcinoma using hyperpolarised [1-^13^C]pyruvate and [1,4-^13^C_2_]fumarate

**DOI:** 10.1038/sj.bjc.6605945

**Published:** 2010-10-05

**Authors:** T H Witney, M I Kettunen, D-e Hu, F A Gallagher, S E Bohndiek, R Napolitano, K M Brindle

**Affiliations:** 1 Department of Biochemistry, University of Cambridge, Tennis Court Road, Cambridge CB2 1GA, UK; 2 Cancer Research UK Cambridge Research Institute, Li Ka Shing Centre, Robinson Way, Cambridge CB2 0RE, UK; 3 Department of Radiology, University of Cambridge, Level 5, Box 218 Addenbrooke's Hospital, Hills Road, Cambridge CB2 0QQ, UK; 4 Department of Chemistry, IFM, via Pietro Giuria 7, Torino 10125, Italy

**Keywords:** tumour, breast cancer, treatment response, pyruvate, fumarate, DNP

## Abstract

**Background:**

The recent introduction of a dynamic nuclear polarisation technique has permitted noninvasive imaging of tumour cell metabolism *in vivo* following intravenous administration of ^13^C-labelled cell substrates.

**Methods:**

Changes in hyperpolarised [1-^13^C]pyruvate and [1,4-^13^C_2_]fumarate metabolism were evaluated in both MDA-MB-231 cells and in implanted MDA-MB-231 tumours following doxorubicin treatment.

**Results:**

Treatment of MDA-MB-231 cells resulted in the induction of apoptosis, which was accompanied by a decrease in hyperpolarised ^13^C label flux between [1-^13^C]pyruvate and lactate, which was correlated with a decrease in the cellular NAD(H) coenzyme pool. There was also an increase in the rate of fumarate conversion to malate, which accompanied the onset of cellular necrosis. *In vivo*, the decrease in ^13^C label exchange between pyruvate and lactate and the increased flux between fumarate and malate, following drug treatment, were shown to occur in the absence of any detectable change in tumour size.

**Conclusion:**

We show here that the early responses of a human breast adenocarcinoma tumour model to drug treatment can be followed by administration of both hyperpolarised [1-^13^C]pyruvate and [1,4-^13^C_2_]fumarate. These techniques could be used, therefore, in the clinic to detect the early responses of breast tumours to treatment.

There has been substantial growth and investment in drug discovery and development programmes in oncology in recent years, with the aim of producing therapeutics targeted at the spectrum of genetic abnormalities found in cancer (Hu *et al*, 2000; Semenza, 2003; Bryant *et al*, 2005). This rapid expansion has moved the field of clinical oncology closer to the realisation of personalised medicine, in which patients receive drugs, individually or in combination, that are tailored to their specific disease. The delivery of this approach should be facilitated by noninvasive imaging methods that allow an early assessment of treatment response, identifying unsuccessful treatments at an early stage and enabling the selection of more effective treatment (reviewed in Brindle (2008a)).

Currently, tumour treatment responses are assessed from reductions in tumour size (Eisenhauer *et al*, 2009). However, this approach lacks sensitivity and many weeks may elapse before a change in size is detected (Neves and Brindle, 2006; Weissleder and Pittet, 2008; Brindle, 2008a). In some cases, for example with cytostatic therapies, there may be no change in size despite a positive response to treatment (Brindle, 2008a). Measurements of tumour physiology or biochemistry, however, can give a much earlier indication of treatment response. Positron emission tomography (PET) measurements of the uptake of the glucose analogue [^18^F] 2-fluoro-2-deoxy-D-glucose (FDG) are being used increasingly in the clinic to stage disease and provide early evidence of treatment response (Eisenhauer *et al*, 2009).

MRS, particularly ^1^H MRS, can also be used to detect the metabolic changes that accompany a positive response to treatment (Aboagye and Bhujwalla, 1999; Glunde *et al*, 2006). The relative lack of sensitivity of MRS, however, limits the spatial and temporal resolution of ^1^H spectroscopic imaging experiments and in many cases only single voxel studies are performed (Hu *et al*, 2009). Although ^1^H MRS experiments are performed widely in the clinic, this lack of sensitivity has limited their routine use. This situation may change with the recent introduction of a dynamic nuclear polarisation (DNP) technique that can increase sensitivity in the solution state ^13^C MRS experiment by >10 000-fold (Ardenkjaer-Larsen *et al*, 2003). The enormous gain in sensitivity means that, following injection of a hyperpolarised ^13^C-labelled cell substrate, there is sufficient signal to image the molecule *in vivo*, and, more importantly, its metabolic conversion into other cell metabolites. Whereas ^1^H MRS measurements provide a largely static picture of the levels of tissue metabolites, this labelling technique enables dynamic imaging of cellular metabolism. The principal drawback of the method, however, is the short half-life of the polarisation; for [1-^13^C]pyruvate this is ∼30 s *in vivo*, which means that the material must be injected and imaged within ∼5 min. Thus, in order to image metabolism using this technique, the hyperpolarised ^13^C-labelled substrate must be taken up rapidly by the cell and its subsequent metabolism must be very fast. Even then, it is often only possible to monitor a single enzyme-catalysed reaction (Gallagher *et al*, 2009a). Nevertheless, the technique has already shown promise for detecting treatment response in tumours (Day *et al*, 2007; Witney *et al*, 2009).

The exchange of hyperpolarised ^13^C label between [1-^13^C]pyruvate and lactate, in the reaction catalysed by lactate dehydrogenase (LDH), was shown to decrease in a drug-treated murine lymphoma *in vivo* (Day *et al*, 2007), where decreased label flux was due to a number of factors, including: DNA damage-mediated activation of polyADP-ribose polymerase (PARP) and consequent depletion of the NAD(H) coenzyme pool, a loss of LDH activity, and a reduction in tumour cellularity (Day *et al*, 2007; Witney *et al*, 2009). A similar study in the same tumour model using [1,4-^13^C_2_]fumarate, showed that the rate of the fumarase catalysed conversion of fumarate to malate was a measure of subsequent drug-induced cellular necrosis (Gallagher *et al*, 2009b). With a clinical trial using hyperpolarised [1-^13^C]pyruvate about to start in prostate cancer (Brindle, 2008b), there is a reasonable expectation that the technique could translate to the clinic, where it offers a new functional imaging approach to detect early tumour responses to treatment.

We show here, in a model of human breast adenocarcinoma, that a combination of hyperpolarised [1-^13^C]pyruvate and [1,4-^13^C_2_]fumarate can be used to detect response to doxorubicin treatment before there is any detectable change in tumour size. There was a decrease in labelled lactate production, reflecting a DNA damage response and initiation of the apoptotic program, and an increase in labelled malate production, reflecting the onset of cellular necrosis.

## Materials and methods

### Cell culture

MDA-MB-231 cells (European Collection of Cell Cultures, Salisbury, Wiltshire, UK) were grown in RPMI 1640 media, supplemented with 10% fetal calf serum, 2 mM L-glutamine, 100 U ml^−1^ penicillin and 100 *μ*g ml^−1^ streptomycin (Invitrogen, Paisley, Refrewshire, UK). Cell number and viability were monitored using trypan blue staining. Cell death was induced by addition of doxorubicin (1 *μ*g ml^−1^ final concentration; Sigma-Aldrich Co. Ltd, Poole, Dorset, UK).

### Flow cytometry

Apoptosis and necrosis were measured by flow cytometry. Cells were trypsinised (0.25% trypsin; 1 mM EDTA) and harvested by centrifugation (1300 **g**, 3 min). Detached cells present in the media before trypsinisation were retained and pooled with the trypsinised cells. Cell pellets (1 × 10^6^ cells) were washed once in ice-cold HEPES-buffered saline (10 mM HEPES, 140 mM NaCl, 2.5 mM CaCl_2_, pH 7.4) and resuspended in 100 *μ*l of the same buffer. Annexin V-Pacific Blue (5 *μ*l per 100 *μ*l of cell suspension) and SYTOX Red (Invitrogen) (5 nM) were added to the cells and incubated for 15 min at 20°C. The resulting mixture was washed once in ice-cold HEPES-buffered saline, kept briefly on ice and then analysed in an LSRII cytometer (BD Biosciences, Rockville, MD, USA), with 20 000 cells counted per event. Viable cells were identified as cells that stain with neither annexin V nor SYTOX Red; apoptotic cells as cells that stain with annexin V, but not SYTOX Red; and necrotic cells as cells that stain with both annexin V and SYTOX Red. Cellular NADH content was assessed from measurements of autofluorescence at 455 nm following excitation at 350 nm (Day *et al*, 2007; Witney *et al*, 2009). Cytosolic NADH has been shown previously to make a significant contribution to this autofluorescence (Aubin, 1979) and it is the cytosolic, rather than the mitochondrial NAD(H) pool, that is depleted the following activation of PARP (Ying *et al*, 2005).

### Western blots

PolyADP-ribose polymerase cleavage was assessed by western blotting as described previously (Witney *et al*, 2009). Membranes were probed using polyclonal rabbit anti-PARP1 antibody (Abcam, Cambridge, Cambridgeshire, UK, 1 : 1000). A rabbit anti-actin antibody (Sigma-Aldrich Co. Ltd, 1 : 1000) was used as a loading control and a peroxidase-conjugated donkey anti-rabbit IgG antibody (Jackson ImmunoResearch Europe Ltd., Newmarket, UK, 1 : 10 000) as the secondary antibody. Proteins were visualised using the ECLplus kit (GE Healthcare, Chalfont St Giles, Bucks, UK). Blots were scanned (PowerLook III, Umax Systems GmbH, Willich, Germany) and signal quantification was performed by densitometry using scanning analysis software (TotalLab TL120, Nonlinear Dynamics, Newcastle upon Tyne, UK).

### [1-^13^C]Pyruvate and [1,4-^13^C_2_]fumarate hyperpolarisation

A 44 mg sample of 91% [1-^13^C]pyruvic acid, containing 15 mM of the trityl radical OXO63 (GE Healthcare, Little Chalfont, UK) and 1.5 mM ProHance (Bracco Diagnostics Inc, Princeton, NJ, USA), was prepared with a 40 mg sample of [1,4-^13^C_2_]fumaric acid dissolved in DMSO, containing 15 mM of the trityl radical AH-111501 and 0.5 mM of a gadolinium chelate, 3-Gd, (GE Healthcare, Little Chalfont, UK). Aliquots (10 *μ*l) of [1,4-^13^C_2_]fumaric acid were dropped into liquid nitrogen to form pellets, which were placed in the DNP polariser with previously frozen [1-^13^C]pyruvic acid. Polarisation was performed using a microwave source at 93.982 GHz and 100 mW for 1 h. The frozen sample was dissolved at 180°C using 6 ml of a 40 mM phosphate buffer, containing 120 mM NaOH and 100 mg l^−1^ EDTA. The dissolved solution contained 20 mM [1,4-^13^C_2_]fumarate and 75 mM [1-^13^C]pyruvate. The sample was dissolved in <5 s and polarisation levels were between 18–25%, determined by comparing the signal intensity from the hyperpolarised sample to that observed at thermal equilibrium (Gallagher *et al*, 2008). For *in vivo* studies, [1-^13^C]pyruvic acid and [1,4-^13^C_2_]fumaric acid pellets were hyperpolarised separately, with 40 mM HEPES, 94 mM NaOH, 30 mM NaCl, 100 mg l^−1^ EDTA and 40 mM phosphate buffer, 40 mM NaOH, 50 mM NaCl, 100 mg l^−1^ EDTA used as the dissolution buffers, respectively.

### ^13^C MRS measurements on cells

Cells (2–6 × 10^7^) were examined in a 10-mm NMR tube, using a broadband probe (Varian NMR Instruments, Palo Alto, CA, USA) in a 9.4-T vertical wide-bore magnet (Oxford Instruments, Oxfordshire, UK) interfaced to a Varian INOVA console (Varian NMR Instruments, Paulo Alto, CA, USA). The sample temperature was maintained at 37°C. Hyperpolarised [1-^13^C]pyruvate (75 mM), hyperpolarised [1,4-^13^C_2_]fumarate (20 mM) and nonhyperpolarised, unlabelled lactate (75 mM) were injected into the cell suspension and single transient ^13^C spectra were acquired every second for 240 s, using a 6° flip angle pulse and a spectral width of 32 kHz. The area under each peak was used to calculate concentration of the labelled metabolites. The two inequivalent malate peaks, ^13^C-labelled at the C-1 and C-4 position, were combined to produce a single measurement of intensity (Gallagher *et al*, 2009b). The peak intensities of hyperpolarised metabolites were fitted to two-site exchange models describing the fluxes of hyperpolarised ^13^C label between pyruvate and lactate (*k*_P_), and fumarate and malate (*k*_F_; Day *et al*, 2007).

### Tumour implantation

Female severe combined immunodeficiency mice were purchased at 6–8 weeks of age from Harlan UK Ltd (Bicester, UK). Tumour cells (1 × 10^7^) were injected subcutaneously into the shaved flanks of mice. Tumour size was measured using callipers and is reported as the product of the two largest perpendicular diameters (mm^2^). Mice were imaged 24 h before treatment with an i.v. injection of 10 mg doxorubicin per kg body weight and at 24 and 48 h post-treatment. All procedures were carried out in accordance with the Animals (Scientific Procedures) Act of 1986 (UK) and were designed with reference to the UK Co-ordinating Committee on Cancer Research Guidelines for the Welfare of Animals in Experimental Neoplasia.

### ^13^C MRS and ^1^H MRI measurements on tumours

Mice were anesthetised with an intraperitoneal injection of 10 ml per kg body weight of a 5 : 4 : 31 mixture of Hypnorm (VetaPharma Ltd, Leeds, West Yorkshire, UK), Hypnovel (Roche, Basel, Switzerland) and saline and a catheter inserted into a tail vein. A 25 mm diameter surface coil tuned to ^13^C (100 MHz) was positioned over the tumour. The entire assembly was placed in a quadrature ^1^H-tuned volume coil (Varian), in a 9.4-T vertical wide-bore magnet. Transverse ^1^H images were acquired from the tumour using a spin-echo pulse sequence (repetition time, 1.5 s; echo time, 30 ms; field of view, 32 mm × 32 mm; data matrix, 256 × 256; slice thickness, 2 mm; 11 slices). Following injection of hyperpolarised [1-^13^C]pyruvate, which was accomplished within 3 s, 128 single transient spectra were collected from a 6 mm tumour slice over a period of 128 s (every sixteenth spectrum was collected from the entire sensitive volume of the surface coil). Spectra were acquired using a slice-selective 600 *μ*s sinc pulse, with a nominal flip angle of 5°. C-1 lactate and pyruvate peak intensities were fitted to the modified Bloch equations for two-site exchange to obtain an apparent rate constant for label exchange between pyruvate and lactate (*k*_P_), as described previously (Day *et al*, 2007). Following [1-^13^C]pyruvate injection, [1,4-^13^C_2_]fumarate was polarised and injected into the animal, which was still anesthetised. This was achieved within 1 h of the previous injection. Owing to the low-malate signals observed, dynamic measurements of fumarate conversion to malate could not be performed *in vivo*. Instead, a single ^13^C spectrum, collected from a 6 mm tumour slice, was acquired 20 s after injection of 0.2 ml fumarate using a nominal flip angle of 20° a time at which the maximum malate peak intensity had been observed in a previous dynamic study in a drug-treated murine lymphoma tumour (Gallagher *et al*, 2009b). Data were expressed as the malate/fumarate ratio, calculated from the intensities of the [1,4-^13^C_2_]fumarate resonance and the combined resonances from [1-^13^C]malate and [4-^13^C]malate.

### Statistics

Results are expressed as the mean±s.d. Significant differences between values were determined using a Mann–Whitney nonparametric test. Statistical analysis was performed using GraphPad Prism version 5.0 for windows (GraphPad Software, San Diego, CA, USA). Differences between treatment groups were considered significant if *P*<0.05.

## Results

### Measurement of cell death following drug treatment *in vitro*

**Figure 1:**
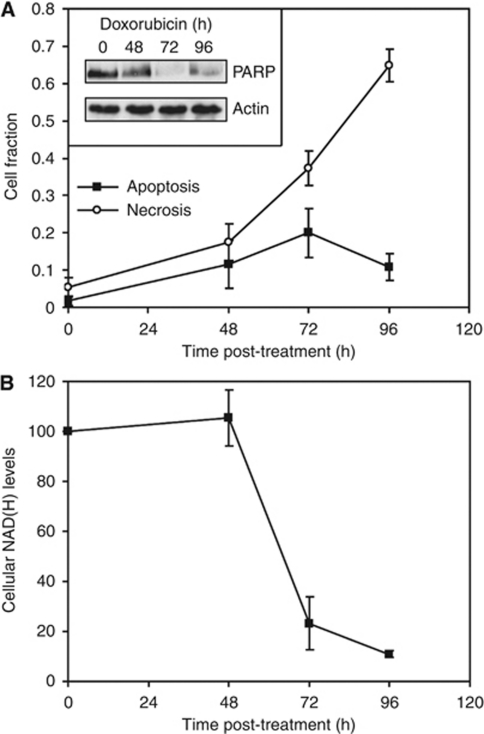
Cell death and NADH depletion in MDA-MB-231 cells following doxorubicin treatment. (**A**) Cell fraction undergoing apoptosis (▪) and necrosis (○), as determined by flow cytometry. Apoptotic cells were detected using annexin V Pacific Blue (*λ* Ex/Em=410/455) and necrotic cells using SYTOX Red nuclear staining (*λ* Ex/Em=640/658). Inset: Representative western blot of PARP expression in MDA-MB-231 cells following doxorubicin treatment. Actin was used as a loading control. (**B**) Changes in the cellular NADH pool following treatment. The NADH pool was measured by recording changes in UV autofluorescence, as detected by flow cytometry (*λ* Ex/Em=350/455).

Drug-induced apoptosis and necrosis of human breast cancer cells (MDA-MB-231) were assessed by flow cytometry, following staining of the cells with Annexin V-Pacific Blue and SYTOX Red, respectively (Figure 1). There were significant increases in apoptosis and necrosis by 48 h after treatment with doxorubicin, which is a commonly used chemotherapeutic drug (Gewirtz, 1999; Figure 1A). Annexin V binding to apoptotic cells correlated with the induction of caspase-3 activity at 48 h, as indicated by PARP cleavage (Kaufmann *et al*, 1993). There was a small decrease in the level of intact PARP at 48 h, with a 26 and 20% decrease compared with cells at 0 h in two independent experiments, with the level of uncleaved PARP becoming almost undetectable at 72 h (Figure 1A, inset). The apoptotic fraction peaked at 72 h, before declining at 96 h, whereas the necrotic fraction was increased at 72 h and further increased at 96 h.

### Monitoring treatment response in cells with hyperpolarised [1-^13^C]pyruvate and [1,4-^13^C_2_]fumarate

**Figure 2:**
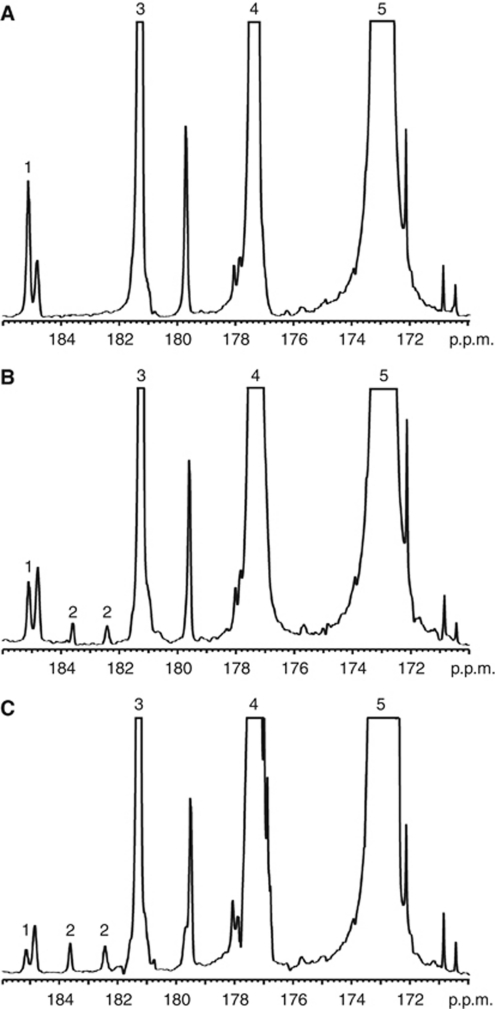
Representative ^13^C spectra of cell suspensions 15 s after the addition of 75 mM hyperpolarised [1-^13^C]pyruvate, 20 mM hyperpolarised [1,4-^13^C_2_]fumarate and 75 mM unlabelled lactate. Cells were either untreated (**A**), or had been treated with doxorubicin for 72 h (**B**) or 96 h (**C**). The labelled peaks are: 1, [1-^13^C]lactate; 2, [1,4-^13^C_2_]malate; 3, [1-^13^C]pyruvate hydrate; 4, [1,4-^13^C_2_]fumarate; 5, [1-^13^C]pyruvate.

**Figure 3:**
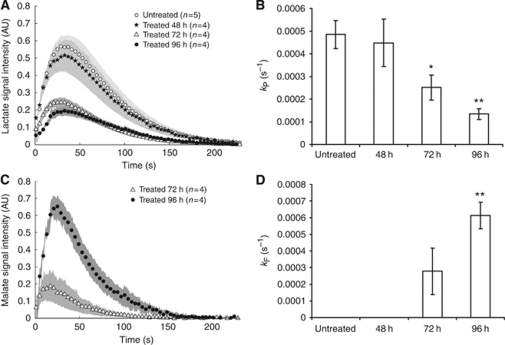
Effect of doxorubicin-induced cell death on flux of hyperpolarised ^13^C label between pyruvate and lactate, and between fumarate and malate in an MDA-MB-231 cell suspension. (**A**) [1-^13^C]lactate peak intensities after addition of 75 mM hyperpolarised [1-^13^C]pyruvate, 20 mM hyperpolarised [1,4-^13^C_2_]fumarate and 75 mM unlabelled lactate to MDA-MB-231 cell suspensions that had been treated with doxorubicin or were untreated. Spectra were corrected for cell number and scaled to the initial pyruvate signal intensity to correct for variation in polarisation levels. Shaded regions represent 1 s.e.m. (*n*=4 for all treatment groups). (**B**) Changes in [1-^13^C]pyruvate–[1-^13^C]lactate exchange rate constant following doxorubicin treatment. The exchange rate constant (*k*_P_) was derived by fitting changes in signal peak intensity to the modified Bloch equations for two-site exchange. Mean values (*n*=4) and s.d. are shown (^*^P<0.05, ^**^P<0.01). (**C**) Total [1,4-^13^C_2_]malate peak intensities after addition of 75 mM hyperpolarised [1-^13^C]pyruvate, 20 mM hyperpolarised [1,4-^13^C_2_]fumarate and 75 mM unlabelled lactate to MDA-MB-231 cell suspensions that had been treated with doxorubicin or were untreated. Spectra were corrected for cell number and scaled to the initial fumarate signal intensity to correct for variation in polarisation levels. Shaded regions represent 1 s.e.m. (*n*=4 for all treatment groups). (**D**) Changes in the rate constant describing hyperpolarised ^13^C label flux between [1,4-^13^C_2_]fumarate and [1,4-^13^C_2_]malate following doxorubicin treatment. The rate constant (*k*_F_) was derived by fitting changes in signal peak intensities to the modified Bloch equations for two-site exchange. Mean values (*n*=4) and s.d. are shown (^*^P<0.05, ^**^P<0.01).

**Figure 4:**
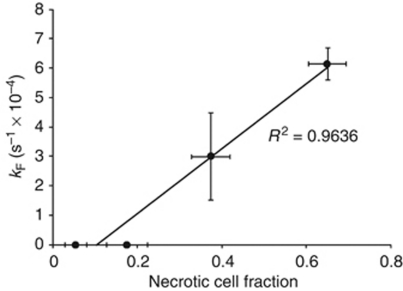
Correlation between cellular necrosis and the rate constant describing hyperpolarised ^13^C label flux between [1,4-^13^C_2_]fumarate and malate. Necrosis was monitored by flow cytometric analysis of SYTOX Red dead cell staining (*λ* Ex/Em=640/658). In separate experiments, the rate constant (*k*_F_) was derived by fitting changes in signal peak intensities to the modified Bloch equations for two-site exchange. Mean values (*n*=3–4) and s.d. are shown.

Addition of hyperpolarised [1-^13^C]pyruvate and [1,4-^13^C_2_]fumarate to MDA-MB-231 cells, resulted in an increase in the lactate carboxyl signal intensity as the hyperpolarised ^13^C label was transferred from pyruvate, followed by a decrease in signal intensity due to decay of the polarisation (Figures 2 and 3). At 72 h after doxorubicin treatment, there was a 48% decrease (*n*=4; *P*<0.01) in ^13^C label exchange between pyruvate and lactate (Figures 2, 3A and B), which was accompanied by a 77% decrease in NADH autofluorescence (*P*<0.01; Figure 1B). At 96 h after drug treatment there was a 72% (*n*=4; *P*<0.01) decrease in label exchange, by which time the NADH autofluorescence had decreased by 89±1% (*P*<0.01). There was no detectable flux of label from hyperpolarised [1,4-^13^C_2_]fumarate to hyperpolarised [1,4-^13^C_2_]-labelled malate until 72 h after doxorubicin treatment (Figure 2), when the apparent rate constant (*k*_F_) for label flux was 2.8 × 10^−4^±1.4 × 10^−4^ s^−1^ (*n*=4). By 96 h, the rate constant had increased by 55% to 6.1 × 10^−4^±0.8 × 10^−4^ s^−1^ (*n*=4; *P*<0.01; Figure 3C and D). There was a good correlation between the extent of cellular necrosis, and the rate constant describing label flux between fumarate and malate (*R*^2^=0.96; Figure 4).

### Monitoring treatment response in tumours with hyperpolarised [1-^13^C]pyruvate and [1,4-^13^C_2_]fumarate

**Figure 5:**
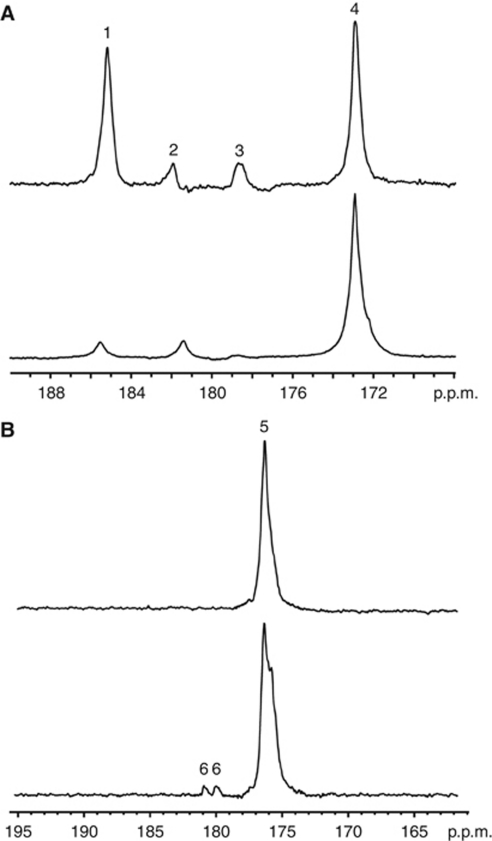
Detection of treatment response in implanted MDA-MB-231 tumours. Representative ^13^C spectra from untreated (upper spectra) or doxorubicin-treated (lower spectra) tumours 20 s post-injection of either hyperpolarised [1-^13^C]pyruvate (**A**), or hyperpolarised [1,4-^13^C_2_]fumarate (**B**). The peaks are: 1, [1-^13^C]lactate; 2, [1-^13^C]pyruvate hydrate; 3, [1-^13^C]alanine; 4, [1-^13^C]pyruvate; 5, [1,4-^13^C_2_]fumarate; 6, [1,4-^13^C_2_]malate. Spectra were taken 48 and 24 h post-doxorubicin treatment for the pyruvate and fumarate experiments, respectively.

Intravenous injection (0.2 ml) of co-hyperpolarised [1-^13^C]pyruvate (75 mM) and [1,4-^13^C_2_]fumarate (20 mM) into MDA-MB-231 tumour-bearing mice resulted in the appearance of signals from [1-^13^C]pyruvate, [1-^13^C]lactate and [1,4-^13^C_2_]fumarate in the tumours. There were no detectable signals from [1,4-^13^C_2_]malate, because of their masking by resonances from labelled lactate and pyruvate hydrate (data not shown). For this reason, [1-^13^C]pyruvate and [1,4-^13^C_2_]fumarate experiments were performed separately. There was a 49% decrease in pyruvate–lactate exchange at 24 h after doxorubicin treatment, where the rate constant (*k*_P_) decreased from 0.075±0.002 s^−1^ in untreated tumours to 0.038±0.025 s^−1^ (*P*=0.15; *n*=3 animals). By 48 h, the rate constant had decreased by 73% to 0.020±0.002 s^−1^ (*P*=0.0017, *n*=3). There was no significant change in tumour size during this period, with the mean tumour volume of 68.0±12.9 mm^2^ (*n*=10 animals) in untreated animals remaining at 65.9±17.3 mm^2^ (*n*=6) and 62.5±16.3 mm^2^ (*n*=6) at 24 and 48 h post-treatment, respectively. Representative spectra from an untreated tumour and a tumour at 48 h after doxorubicin treatment are shown in Figure 5A. The maximum amplitude of the [1,4-^13^C_2_]malate signals relative to the signal from [1,4-^13^C_2_]fumarate 20 s post-injection increased from 0.017±0.013 in untreated tumours to 0.090±0.050 at 24 h after doxorubicin treatment (*n*=4; *P*<0.05), a 5.4-fold increase (Figure 5B).

## Discussion

Imaging currently has an important role in the management of breast cancer patients by enabling assessment of treatment response following primary or neoadjuvant therapy. Therapeutic response is assessed primarily by measurements of tumour shrinkage, using a combination of computed tomography (CT) and/or MRI (reviewed in Ollivier *et al* (2005)). However, there are problems with these imaging modalities. CT has the advantages of simplicity, speed and availability, however, changes in tumour size are frequently overestimated in diffuse or multinodular tumours (Ollivier *et al*, 2005). Changes in tumour size can also be overestimated by MRI, particularly with smaller tumours and if there is surrounding tissue oedema (Merchant *et al*, 1993). Dynamic contrast-enhanced MRI can provide further diagnostic information, with the intensity of contrast enhancement shown to predict the presence of residual tumour after preoperative chemotherapy (Gilles *et al*, 1994). There is a need, however, to develop imaging methods that can detect treatment response more sensitively and before there is any change in tumour size.

FDG-PET is being used increasingly in the clinic as a functional imaging technique for detection, staging and early response monitoring in breast cancer (Eubank and Mankoff, 2005). Although currently most useful as a staging tool, initial studies have shown FDG-PET to be capable of predicting pathological response in patients with locally advanced breast cancer following a single course of neoadjuvant chemotherapy (Biersack *et al*, 2004; Krak *et al*, 2004). However, the presence of infected or inflamed tissue, and high uptake by infiltrating immune cells can mask changes in tumour FDG uptake (Strauss, 1996; Zhuang *et al*, 2005). Patients also receive a high radiation dose, with scans being costly, time-consuming and are only available in a few selected centres. Complementary methods for the detecting treatment response in breast cancer are therefore still required that provide alternative biological readouts that can be used in conjunction with existing methods in a multimodality approach.

Treatment of murine EL-4 lymphoma cells with a topoisomerase II inhibitor and DNA damaging agent, etoposide, was shown previously to inhibit the rate of hyperpolarised ^13^C label exchange between [1-^13^C]pyruvate and lactate, catalysed by the enzyme LDH. The decreased exchange was explained by induction of a DNA damage response and consequent induction of PARP, for which the coenzyme NAD^+^ is a substrate (Day *et al*, 2007; Witney *et al*, 2009). We have shown here that doxorubicin treatment of the oestrogen receptor negative-human breast cancer cell line, MDA-MB-231, *in vitro* resulted in a marked decrease in the rate of hyperpolarised ^13^C label exchange between [1-^13^C]pyruvate and lactate and that this was correlated with a decrease in the concentration of the cellular NAD(H) coenzyme pool. Doxorubicin, like etoposide, is a topoisomerase II inhibitor and a DNA damaging agent that induces PARP activation (Munoz-Gamez *et al*, 2005). Therefore, this hyperpolarised [1-^13^C]pyruvate method for detecting response to genotoxic agents would appear to be generally applicable to other tumour types.

The hyperpolarised [1-^13^C]pyruvate experiment shows that the tumour cell has responded to treatment, but not that it has died. Moreover, it provides ‘negative contrast’, in that there is a decrease in lactate signal intensity in those tumours that have responded to treatment (Day *et al*, 2007; Witney *et al*, 2009). Such a decrease in lactate signal could also be due to other factors, such as reduced delivery of pyruvate to the tumour. In contrast, hyperpolarised ^13^C-labelled [1,4-^13^C_2_]fumarate provides positive contrast and appears to be a marker of cell death (Gallagher *et al*, 2009b). Fumarate shows relatively slow plasma membrane transport, and thus, in viable cells there is no detectable conversion of fumarate to malate within the relatively short lifetime of the ^13^C polarisation. However, in necrotic cells, where the plasma membrane permeability barrier has been compromised, fumarate is rapidly converted to malate and the hyperpolarised [1,4-^13^C_2_]malate signal provides a positive marker of tumour cell necrosis. Treatment of MDA-MB-231 cells with doxorubicin resulted in the production of significant levels of labelled malate by 72 h after drug treatment, a time at which there was also a significant decrease in the rate of labelled lactate production. There was no detectable labelled malate produced in untreated cells. The rate of labelled malate production showed a good correlation with the level of tumour cell necrosis, as has been observed previously (Gallagher *et al*, 2009b). As with the hyperpolarised [1-^13^C]pyruvate method for detecting response to genotoxic agents, these experiments have demonstrated that the hyperpolarised [1,4-^13^C_2_]fumarate experiment could be a generic method for detecting tumour cell death post-treatment.

Experiments with hyperpolarised [1-^13^C]pyruvate and [1,4-^13^C_2_]fumarate were also performed in implanted MDA-MB-231 tumours, where we have shown a response to doxorubicin treatment in the absence of any detectable change in tumour size. Co-hyperpolarised [1-^13^C]pyruvate and [1,4-^13^C_2_]fumarate preparations could not be used *in vivo* as malate production was masked by overlapping signals from lactate and pyruvate hydrate formed from the labelled pyruvate in these lower resolution ^13^C spectra. When the pyruvate was injected alone, a significant decrease in pyruvate–lactate exchange was observed at 48 h post-therapy at the maximum tolerated dose of 10 mg kg^−1^ doxorubicin. This relatively slow response to therapy is consistent with that observed in cultured cells and reflects the relative resistance of MDA-MB-231 tumours to genotoxic agents (Fang *et al*, 2007). At 24 h, after treatment with doxorubicin and 20 s after the injection of hyperpolarised [1,4-^13^C_2_]fumarate the hyperpolarised ^13^C malate/fumarate ratio increased 5.4-fold (Figure 5B), indicating the onset of tumour cell necrosis. At this time point, a decrease in pyruvate–lactate exchange was also observed, but did not reach significance. Therapy response was observed earlier in tumours than in the cell experiments, with the larger number of tumour cells *in vivo* giving an increase in sensitivity.

The level of tumour cell death after drug treatment has been shown, in preclinical and clinical studies, to be a good prognostic indicator for treatment outcome, including in breast cancer (Chang *et al*, 2000). However, early evidence of treatment response does not necessarily indicate a favourable long-term outcome. For example, an early decrease in FDG uptake in oesophageal adenocarcinoma in the clinic following neoaduvant treatment did not correlate with a histopathological response in the longer term (Smithers *et al*, 2008) and in head and neck cancers treated with radiotherapy uptake of l-[methyl-^11^C]methionine was reduced, but there was no correlation with treatment outcome (Nuutinen *et al*, 1999). However, in the case of the MDA-MB-231 human breast cancer model used in this study, a single treatment with doxorubicin, at a slightly lower dose than used here, has been shown to produce a long-term treatment response, as indicated by a tumour growth delay of ∼20 days (Mason *et al*, 2008). Thus, the imaging methods that we have described here can detect early evidence of a treatment response that is correlated with outcome in the longer term.

In summary, we have shown that hyperpolarised ^13^C experiments for detecting early evidence of treatment response, which had previously only been demonstrated in an implanted murine lymphoma tumour model that responds very rapidly to treatment with a genotoxic agent (Day *et al*, 2007; Witney *et al*, 2009), also work in a human breast tumour xenograft that is much more resistant to treatment with these agents. The hyperpolarised [1-^13^C]pyruvate experiment provided early evidence of a DNA damage response, whereas the [1,4-^13^C_2_]fumarate experiment indicated the presence of tumour cell necrosis post-treatment. Both experiments indicated the presence of a treatment response in the absence of any change in tumour size. Translation of this technique to the clinic could offer a new method for monitoring treatment response in breast cancer.
